# Lack of neophobic responses to color in a jumping spider that uses color cues when foraging (*Habronattus pyrrithrix*)

**DOI:** 10.1371/journal.pone.0254865

**Published:** 2021-07-29

**Authors:** Michael E. Vickers, Madison L. Heisey, Lisa A. Taylor

**Affiliations:** 1 Entomology and Nematology Department, University of Florida, Gainesville, FL, United States of America; 2 Department of Zoology and Entomology, University of the Free State, Bloemfontein, Republic of South Africa; 3 Florida Museum of Natural History, University of Florida, Gainesville, FL, United States of America; Liverpool John Moores University, UNITED KINGDOM

## Abstract

Chemically defended prey often advertise their toxins with bright and conspicuous colors. To understand why such colors are effective at reducing predation, we need to understand the psychology of key predators. In bird predators, there is evidence that individuals avoid novelty—including prey of novel colors (with which they have had no prior experience). Moreover, the effect of novelty is sometimes strongest for colors that are typically associated with aposematic prey (e.g., red, orange, yellow). Given these findings in the bird literature, color neophobia has been argued to be a driving force in the evolution of aposematism. However, no studies have yet asked whether invertebrate predators respond similarly to novel colors. Here, we tested whether naive lab-raised jumping spiders (*Habronattus pyrrithrix*) exhibit similar patterns of color neophobia to birds. Using color-manipulated living prey, we first color-exposed spiders to prey of two out of three colors (blue, green, or red), with the third color remaining novel. After this color exposure phase, we gave the spiders tests where they could choose between all three colors (two familiar, one novel). We found that *H*. *pyrrithrix* attacked novel and familiar-colored prey at equal rates with no evidence that the degree of neophobia varied by color. Moreover, we found no evidence that either prey novelty nor color (nor their interaction) had an effect on how quickly prey was attacked. We discuss these findings in the context of what is known about color neophobia in other animals and how this contributes to our understanding of aposematic signals.

## Introduction

Aposematic prey are very diverse and how this diversity evolves is a question that has garnered considerable interest over the years [1]. It is largely recognized that understanding this diversity requires understanding the psychology of key predators [1–3]. This is exemplified by the vast bird literature that has examined everything from innate responses to colorful prey, to how color affects a predator’s learning process, to how different components of a prey’s defenses (including color) interact to influence predation [e.g., 4–22].

One interesting area of this work includes the caution that many predators show towards novel-colored prey. ‘Dietary wariness’ includes an initial avoidance that some predators show towards novel prey (termed ‘neophobia’) and sometimes also a longer-term hesitancy to incorporate such foods into their diet (termed ‘dietary conservatism’) [7]. When such responses result from novel colors in prey, these processes have been argued could be a driving force in the evolution of aposematism [9, 19, 23, 24]. When presented with colorful food, birds and other animals often show avoidance of novel-colored prey compared with prey of familiar colors [4, 6–10]. Interestingly, avian predators sometimes show a stronger avoidance of colors typically associated with aposematism (such as red and yellow) compared to other colors (e.g., [19]; but see [9]). While these patterns have been well-documented in birds, the extent to which they also exist in other predators is unclear.

There are many terrestrial invertebrates that feed on insects [25]; while the visual systems of these predators vary widely, many include some degree of color vision [26]. Despite this widespread ability to use color cues in foraging decisions, we know of no studies on color neophobia in any invertebrate predator. What we do know about similar phenomena in invertebrates comes from non-predators that distinguish between novel and familiar colors in contexts other than predation. For example, foraging bumblebees exposed to novel flower colors have longer latencies to feed compared to familiar-colored flowers [27, 28]. This suggests that a tendency to avoid novel colors may also be a feature of invertebrate predators’ hunting behavior but has yet to be examined.

Here, we examined patterns of neophobia towards novel-colored prey in naïve lab-raised *Habronattus pyrrithrix*, a jumping spider that recent work suggests has trichromatic vision, including the ability to see and discriminate long-wavelength colors that are common in aposematic displays [29]. *Habronattus* jumping spiders are a particularly interesting group to examine color neophobia. *Habronattus* is a diverse genus with members that are common in a variety of habitat types across North America [30]; as such they are likely to encounter a variety of colorful and chemically-defended prey items in the field. The chemical defenses of many insects are unpalatable and/or toxic to *Habronattus* [e.g., 31–33], so it is not surprising that these spiders attend to color when foraging. What has been surprising is how similar their responses to color are to those of birds; multiple recent studies have shown that *Habronattus* and other jumping spiders have similar patterns of innate and learned color biases to birds [31, 34–37], similar patterns of color learning and generalization [31, 35], and similar color aversions that are triggered by noxious odors [38, 39]. Understanding whether these similarities with birds extends to color neophobia will provide insights into broad patterns of predator psychology that are shared across distantly-related taxa.

Based on the patterns observed in the literature for other visual predators (primarily birds) [4, 6–10] we hypothesized *a priori* that spiders would show a neophobic response towards novel-colored prey. Also following patterns in the avian literature [19], we hypothesized *a priori* that any neophobic response to color would be stronger for certain colors (specifically, colors that are commonly used as aposematic signals) compared with others. To test these two hypotheses, we first exposed different subsets of spiders to different combinations of prey colors (including red, green, and blue) by feeding them with artificially-colored termites for four weeks. We then used choice tests with these termites to ask if spiders would be more likely to attack prey of familiar colors (to which they had previously been exposed) vs. a novel color (to which they had never been exposed). We also asked whether any effect of novelty would be stronger when the novel color was red (as red is typically associated with aposematic prey), compared with when the novel colors were green or blue (which are less commonly associated with aposematic prey). Alongside these tests of our focal hypotheses, we also explored whether there were any innate color biases that were stable enough to persist through the four-week color exposure phase of our study, and may have influenced our results. Despite much interest in the role of neophobia in shaping responses to colorful prey in animals, this is, to our knowledge, the first study to examine the specific role that neophobia plays in responses to prey colors in any invertebrate predator.

## Materials and methods

### Collection and maintenance of spiders

We collected *H*. *pyrrithrix* adult females in 2018 and 2019 from a single population in Queen Creek, AZ, USA and housed and maintained them using previously published methods [34]. They were allowed to lay egg sacs, and once spiderlings emerged, we separated these spiderlings into individual plastic snap-cap vials (25 mm dia x 70 mm H). The spiderlings were fed approximately their own mass in newly hatched crickets (*Gryllodes sigillatus*) 3x per week. Once spiders reached approximately 4 mm in length, we determined their sex using color patterns [40] and retained only juvenile females (n = 128) for the study. To maximize genetic diversity, we used spiderlings from 41 individual mothers (with no more than 10 spiderlings from any one mother). Because these females were entirely lab-raised and fed only crickets, they were naive to the prey colors used in our experiment (red, green, and blue). Previous work suggests these spiders have prey color biases with both innate and learned components (with the highest attack rate on blue and the lowest on red and yellow) [34, 36]; we consider the possible effects of these biases in our analyses and discussion (see below).

### Artificially-colored prey

**Fig 1:**
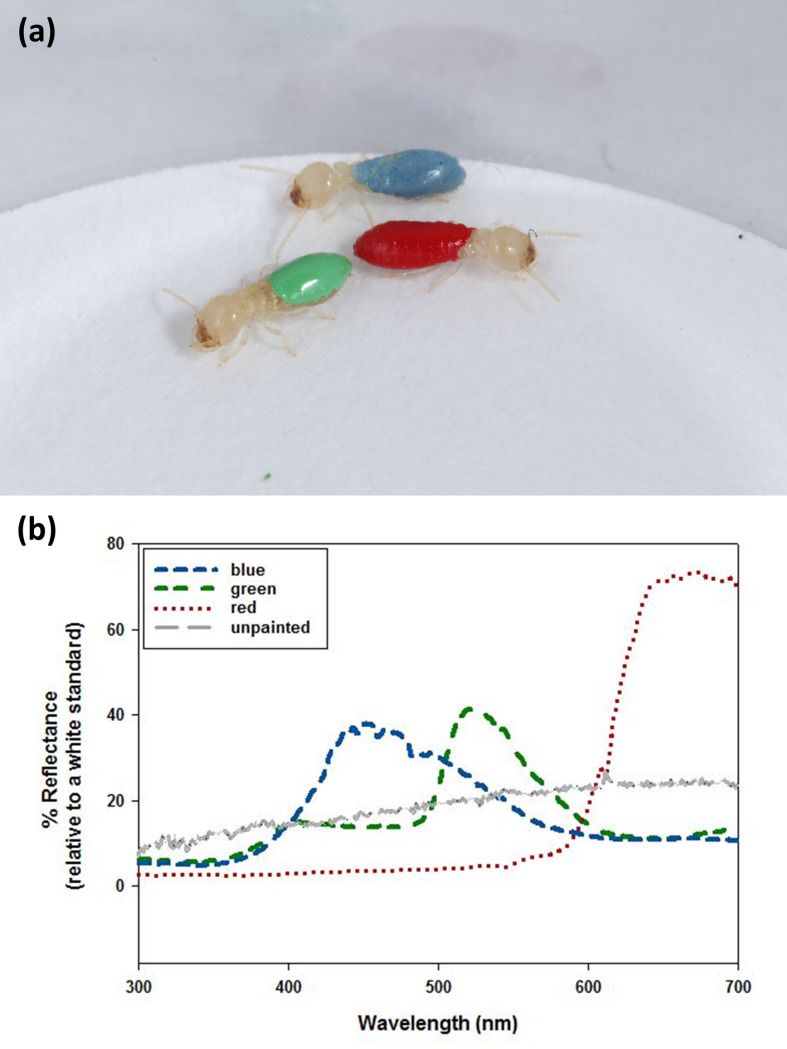
(a) Termites with abdomens painted blue, green, or red (Photo credit: Lyle Buss), (b) spectral properties of enamel paints (either blue, green, or red) used to paint termites for color choice tests. The spectral properties of unpainted (naturally colored) termites are shown for comparison. For these color measurements, each paint color was applied to filter paper and allowed to dry for 1 hr. Once dry, we used a UV-vis spectrophotometer (USB 2000 with PX-2 pulsed xenon light source, Ocean Optics, Dunedin, FL, USA) to collect spectral data. During measurements, the spectrophotometer probe was held perpendicular to the colored surface. We used a measurement pin to ensure a consistent distance between the probe and sample. Spectral readings were taken relative to a Spectralon diffuse reflectance white standard (Labsphere Inc., North Sutton, NH, USA). The spectral curves shown represent the mean of 10 measurements for each color.

We collected termites (*Reticulitermes flavipes*) from the Natural Area Teaching Lab at the University of Florida, Gainesville, FL, USA and maintained them in the lab for the duration of the project. We artificially colored them by painting their dorsal abdomens using enamel paint following methods previously used to study jumping spider prey color preferences [32, 36, 38, 39] (Fig 1). For this experiment, we used three colors: blue, green and red, that were similar in brightness. While our understanding of the *Habronattus* visual system is still in its infancy [29], keeping the brightness consistent between the three colors across the jumping spiders’ visible range increased the likelihood that any differences in response that we see will be due to chromatic rather than achromatic (i.e., brightness) cues. To standardize brightness across the three paint colors, we used unmanipulated red paint (Testor Corporation, Rockville, IL, USA, product number: 1150-RM11501-0611), and adjusted the brightness of green (product number: 1124-RM11241-0611) and blue (product number: 1176-RM11761-0711) paint by adding white (product number: 1168-RM11681-0611) until all three paint colors had equal brightness. Following methods by Montgomerie [41], we calculated brightness as the mean reflectance across the visible spectrum (i.e., the range that evidence suggests is visible to jumping spiders, 280–700 nm, see [42, 43]) and found no significant differences among the three colors (*F*_2,421_ = 0.06, *p* = 0.94; Fig 1). Before using the painted termites for experiments we allowed them to dry for one hour. The enamel paint does not emit any noticeable odor; however, because all termites used during the study were painted (either blue, green, or red), any residual paint odor remaining after the drying period should be equivalent across all termite colors. Prior research has shown that enamel paint on termites does not affect their movement rates when compared to unpainted termites [38]. We chose to use termites as prey in this study because they are palatable prey items that are readily attacked and consumed by the spiders, their colors are easy to manipulate, and they have been used successfully as prey in several previous studies with *Habronattus* jumping spiders [32, 36, 38, 39].

### Color exposure phase of experiment

We began the experiment with a color exposure phase, where each spider (n = 128, still immature and approx. 4mm in length) was fed painted termites of two randomly selected colors (from the pool of three colors) three times per week for a period of four weeks. At each feeding, we fed these spiders two painted termites (with each termite being approximately equivalent to their own body length, as determined by visual inspection). This training prey was the only food that the spiders received during this 4-week period; we observed each spider during their first feeding to confirm that every test spider attacked and consumed termites. Because the spiders were repeatedly exposed to these two colors at every feeding, they will hereafter be referred to as ‘familiar colors’. The third color was assigned to be a novel color; spiders had no experience with this color during the color exposure phase of the experiment. We fed spiders in round (9 cm diameter) arenas (petri dishes) lined with white filter paper to provide a consistent visual background; the spiders remained in these arenas for the duration of the color exposure phase of the study. Before each feeding, we removed any old prey remaining from the previous feeding. Because color vision in *Habronattus* jumping spiders seems to be light-limited [29, 44], we fed and maintained spiders under full-spectrum artificial lights (SoLux MR16 3500K 50W and SoLux PAR38 3500K 90W, Tailored Lighting Inc, Rochester, NY, USA) supplemented with additional natural sunlight from two large windows directly adjacent to our feeding area. This setup allowed us to maximize access to natural light, while also ensuring that the spiders received sufficient additional light on cloudy or overcast days. We began all feedings during daylight hours (between 0900 and 1700 hours).

### Choice tests between familiar and novel-colored prey

After the four-week color exposure period ended, we gave each spider a test where they could choose between three termites (one of each of the three colors). Two of these colors were familiar (as they had been exposed to these colors during the color exposure phase of the study) and the third color was novel (i.e., they had never been exposed to it). We ran these choice tests within one day of each spiders’ last color exposure feeding; jumping spiders have relatively short-term memories (a detail that we address further in the Discussion, see [31, 45]), so we designed this testing schedule to increase the likelihood that the spiders would retain memories of their color exposure feedings.

For choice tests, we presented the colored termites to the spiders in the same arenas where their regular feedings occurred (described above). As with their regular feedings, the termites were freely moving in the arenas during the choice tests. Before the spiders were allowed to attack any termites in the choice tests, we first gave them a 10-minute acclimation period where we placed them in a round 3.5 cm clear inner chamber in the center of the arena; this allowed the spiders to view the colored termites and see that there were multiple prey options before attacking. Jumping spiders respond to stimuli, including prey, by quickly orienting their large forward-facing anterior median eyes toward a target [e.g., 46]. During the acclimation period, we observed the spiders and confirmed that, in every trial, the test spider oriented to each of the three colors at least once. After the acclimation period, we removed the inner chamber lid, allowing the spiders to exit and freely capture the termites. If the spider did not exit the acclimation chamber within 5 minutes, the trial was stopped and the spider was not retested. When the spider exited the acclimation chamber, we recorded the color of the termite that the spider first captured; the trial ended immediately after this occurred. We also recorded the time of this attack (as a measure of attack latency). If no termite was captured for 15 minutes, the trial ended, and the spider was not re-tested. No spiders were used in more than one test.

### Statistical analyses

To assess (1) whether the spiders were less likely to attack novel-colored prey (compared with familiar-colored prey), (2) whether the spiders showed robust innate color biases that persisted through the 4-week color exposure period, and (3) whether any effects of novelty differed between colors, we took two different statistical approaches (each with different benefits and limitations, described below). Data for these analyses came from the 76 out of 128 trials in which the spider successfully attacked a termite; since our analyses focus on comparing attacks rates, we excluded trials where no attack was made within the 15 minute trial time allotted.

First, we used a generalized linear mixed model (GLMM), with a binomial distribution (logit function). The fixed effects were the color of the termites (either blue, green, or red), whether or not those colors were novel to the spider in the trial (Y/N), and the interaction of color and novelty. The response variable was whether or not each termite in a trial was attacked. Because each spider was presented with three termites during a trial, trial ID was included as a random effect. The interaction of color and novelty allowed us to assess whether any effects of novelty differed among the three colors. If there was no interaction, the main effect of color allowed to assess whether the spiders had any persistent innate color biases. And the main effect of novelty allowed us to assess whether the spiders were avoiding novel-colored prey (compared with prey of familiar colors). This was the most straightforward way to simultaneously assess the main effects of novelty and color, as well as their interaction, in a single model that takes all factors into account together. However, this approach also has one limitation. Specifically, the data are autocorrelated; the spider was only allowed to attack one of the three termites, so if they attacked one color, they did not attack the other two. This issue increases the risk of type 1 error and therefore any positive results uncovered with this method should be interpreted with caution.

Due to the concern with the GLMM described above, we also addressed the same three questions with additional follow-up *X*^*2*^ analyses of the same data. The limitation of this second approach is that it only allows us to examine the main effects of color and novelty separately, leaving us unable to statistically assess any interaction; this is the reason that we ran both the GLMM and the *X*^*2*^ analyses. First, to assess whether the spiders were less likely to attack novel-colored prey (compared with familiar-colored prey), we used a likelihood ratio *X*^*2*^ test to ask whether the rate of attack on novel-colored prey items was different than would be expected by chance alone. Because each test consisted of an individual spider being presented with one novel-colored and two familiar-colored prey items, we expect twice as many spiders to attack familiar-colored prey due to chance alone. Second, to assess whether any effect of novelty differed by color, we analyzed the data separately depending on whether the novel color in the trial was blue, green, or red. For each of these cases, we analyzed the data in the same way as above, asking whether the color was attacked at lower rates when it was the novel color (compared with the familiar colors). Here, we predicted *a priori* that the blue and green would be attacked at the same rates as the others, even when they were novel. In contrast, we expected that red (a color commonly used in aposematic displays) would be attacked at lower rates when it was novel. After finding no effect of neophobia for any of the colors, we went on to explore whether there was any evidence of persistent color biases (that had persisted through the training phase of our experiment). For this, we used a likelihood ratio *X*^*2*^ test to ask whether there were differences in attack rates among the three colors.

In the absence of any evidence of color neophobia in our analyses of which colored termites were attacked (see Results), we went on to explore attack latency. Specifically, we asked (1) whether spiders were quicker to attack novel-colored prey than familiar-colored prey, (2) whether spiders were quicker to attack certain colors over others, or (3) whether any effects of novelty on the latency to attack differed between the three colors, using ANOVA. The fixed effects were color, novelty, and the interaction of color and novelty. The response variable was the time (seconds) it took the spider to attack.

Given that spiders attacked termites in only 76 out of 128 trials, we conducted an exploratory analysis (likelihood ratio *X*^*2*^ test) to consider whether the trial success rate (i.e., whether the test spider successfully attacked a termite) differed depending on which color was novel. While we had no *a priori* expectations that this would be the case, unexpected differences in success rates across these groups might suggest that the spiders were less motivated to hunt at all when presented with certain color/novelty combinations.

All analyses were conducted using SPSS Statistics (version 26) and JMP Pro 15.

## Results

**Fig 2:**
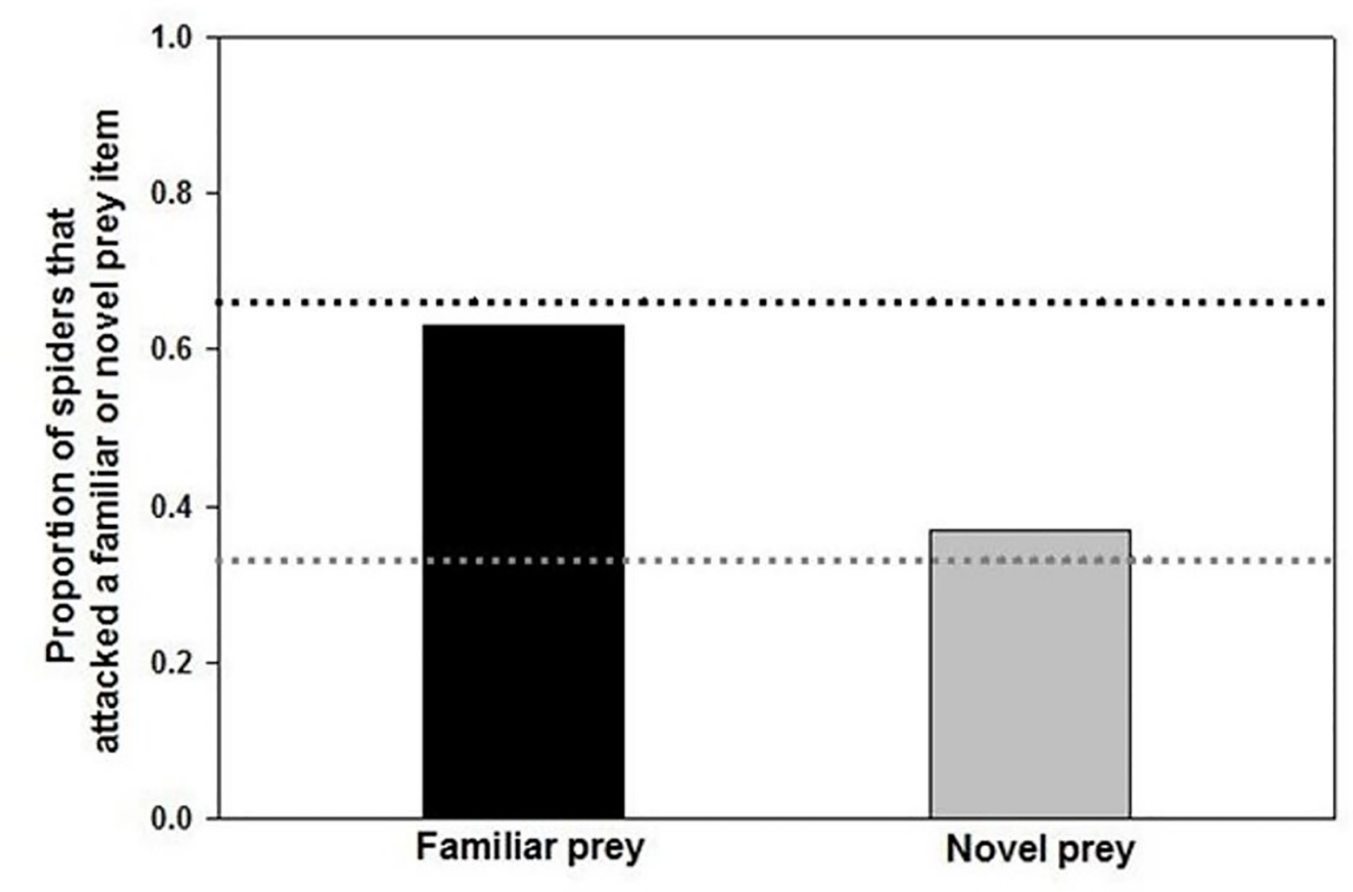
The proportion of *Habronattus pyrrithrix* spiders that attacked either a familiar- or novel-colored prey item during choice tests. Because each test consisted of an individual spider being presented with one novel-colored and two familiar-colored prey items, we expect twice as many spiders to attack familiar-colored prey due to chance alone. The black dotted line indicates the proportion of spiders expected to attack familiar colors and the gray dotted line indicates the proportion of spiders expected to attack novel-colored prey items, due to chance alone. The results shown here do not differ from chance.

**Fig 3:**
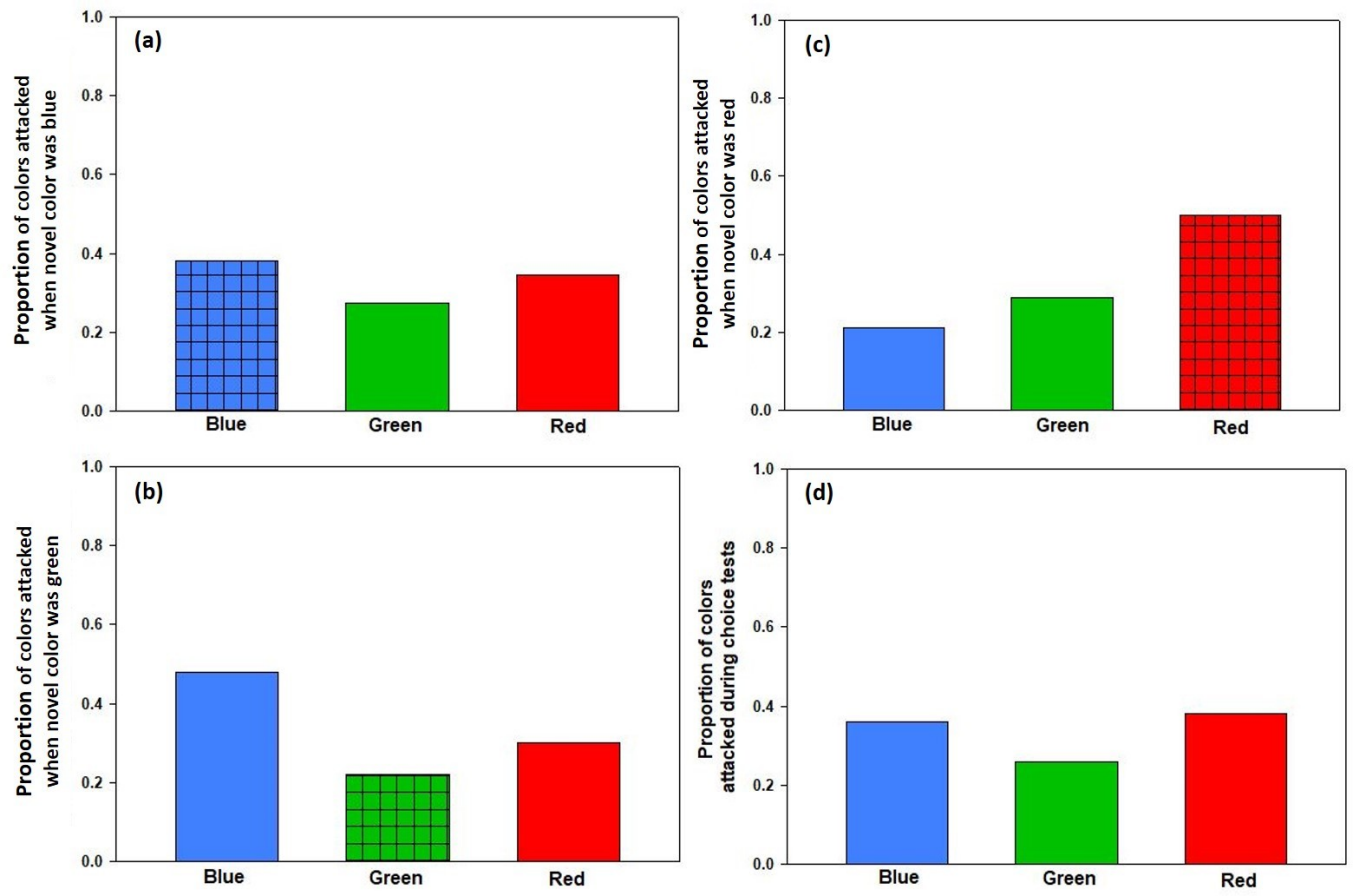
The proportion of colors attacked by *Habronattus pyrrithrix* spiders in choice tests when the randomly assigned novel color was blue (a), green (b), and red (c). The lack of a significant color*novelty interaction indicates that any effect of novelty did not differ among the three treatment groups. The proportion of colors attacked overall (with data pooled) is shown in (d). Checkered patterns on the bars indicate which color was novel during trials.

Neither the color of a termite, whether that color was novel or familiar, nor the interaction between color or novelty, predicted whether a termite would be attacked in a trial (Table 1). The lack of a significant color*novelty interaction suggests that any effect of novelty did not differ among the three colors (Fig 3). These data suggest no effect of neophobia on the colors that the spiders attacked, as the spiders attacked novel and familiar-colored termites at the same rates as would be expected by chance alone (Fig 2). In addition, these data suggest that any innate color biases did not persist through the color exposure phase of the experiment, as the spiders attacked the three different colors of termites at equal rates (Fig 3).

**Table 1:** Generalized linear mixed model (GLMM) results examining effects of color, novelty, and their interaction on whether or not a termite was captured by lab-raised *Habronattus pyrrithrix* during choice tests.

Fixed Effect	df	*F*	*P*
Prey color	2,222	1.90	0.15
Novelty	1,222	0.34	0.56
Prey color*Novelty	2,222	0.97	0.38

Our follow-up *X*^*2*^ analyses revealed the same patterns as those uncovered with the GLMM. There was no evidence of color neophobia: spiders attacked novel- and familiar- colored prey at the same rate as expected by chance (*X*^*2*^ = 0.41, *P* = 0.52, Fig 2). We also found no evidence that any effect of neophobia differed by color in the ways predicted by current theory. Whether the novel color was blue, green, or red, spiders attacked the novel color at the same rate as would be expected by chance (blue: *X*^*2*^ = 0.27, *P* = 0.60; green: *X*^*2*^ = 1.50, *P* = 0.22; red: *X*^*2*^ = 2.83, *P* = 0.09; Fig 3A–3C). Note that the non-significant trend seen when red is novel is opposite expectation: when red was the novel color, the spiders tended to attack it *more* than the familiar colors (rather than less). Finally, we found no evidence of persistent color biases. When all of the data were pooled, there were no differences in attack rates on the three colors (*X*^*2*^ = 1.83, *P* = 0.42; Fig 3D).

We found that neither the termite color, whether the color was novel or familiar, nor the interaction between color and novelty predicted how long it would take spiders to attack (Table 2).

**Table 2:** ANOVA results examining effects of color, novelty, and their interaction on the time lab-raised *Habronattus pyrrithrix* took to attack termite prey during choice tests.

Fixed Effect	df	*F*	*P*
Prey color	2,70	0.75	0.48
Novelty	1,70	1.70	0.20
Prey color*Novelty	2,70	0.03	0.97

In our exploratory analyses, the success rate of trials did not differ depending on which color was novel (*X*^*2*^ = 0.95, *P* = 0.62; when blue was novel: 29 successful, 17 unsuccessful; when green was novel: 23 successful, 20 unsuccessful; when red was novel: 24 successful, 15 unsuccessful).

## Discussion

We began this study with two clear *a priori* hypotheses informed by a large body of literature on predator psychology; this literature has mostly been built with empirical studies using birds. First, we hypothesized that, like birds, *Habronattus pyrrithrix* jumping spiders would show a neophobic response towards novel-colored prey [4, 6–10]. Second, we hypothesized that, like birds, the degree of neophobia would be stronger for some colors (e.g., those typically associated with aposematism) compared to other colors [19]. However, we found no support for either of these hypotheses. Here we show that lab-raised *H*. *pyrrithrix* attacked novel and familiar-colored prey at equal rates with no evidence that the degree of neophobia varied by color. Moreover, we also found no evidence that either novelty nor color (nor their interaction) affected the time it took spiders to attack prey. Aside from our unexpected results in the present study, other recent work with jumping spiders and colorful prey has shown striking similarities with birds. This is despite their small size, and markedly different visual systems and brains. Jumping spiders show similar patterns of innate and learned color biases to birds, often avoiding certain prey colors, such as red and yellow, which are typically associated with aposematism [e.g., 31, 34–36]. They show similar patterns of color learning to birds, with only subtle differences in the degree of color generalization [31, 35]. They have color aversions that are predictably triggered by noxious odors, in much the same way as birds [38, 39]. All of these similarities are what made us expect that these spiders would also have similar patterns of color neophobia to those seen in birds.

Why might color neophobia that is so prevalent in bird predators be absent in these jumping spiders? It may be that there are key differences between these two taxa that result in different costs and benefits of neophobic responses to color. For example, previous work shows that jumping spiders have relatively shorter memories when learning about colorful prey; learned aversions to colorful and unpalatable prey last anywhere from a few hours to two weeks, depending on how many interactions they have with the prey [31, 45]. By contrast, birds’ aversions to colorful prey can last substantially longer (ranging from a few months to over a year, reviewed in [47]). The shorter-term memory of jumping spiders may mean that they will more often encounter prey that is ‘novel’, simply because they do not remember their prior interactions with it. This may make general neophobic responses to color less useful and particularly costly leading to missed predation opportunities in the field. Learning is undoubtedly important for jumping spider predators [48]; future work could use mathematical models to help us understand how the limits of memory might influence the costs and benefits of neophobia.

Another major difference between these two taxa is that insectivorous birds typically feed on prey that is much smaller than themselves [49] compared with jumping spiders that will regularly attack insect prey that is up to twice their own body size [50]. This likely makes hunting inherently riskier for jumping spiders compared to most birds. Once jumping spiders decide to attack, they often leap at their prey, grapple with it and envenomate it to subdue it [51, 52]. If that prey is defended with a sting or bite, the encounter could be deadly. Even the termite prey used in the present study were large enough to be considered risky, as they were approximately the same size as the test spiders. As a result of this large size, the spiders may have shown equal caution with all of the termites and may have been less likely to reserve caution for just those of novel colors. Because our study is the first to examine color neophobia in any jumping spider, it is too soon to say how generalizable our findings may be. Future work could manipulate the size of colorful prey choices (perhaps using smaller termites or small hemipterans or flies, e.g., [32]) or other aspects of prey riskiness. In addition, manipulating other factors that affect the motivation of predators (e.g., hunger level, previous experience with defended prey, etc.) may reveal patterns of neophobia that were not uncovered in our experiment.

When reporting negative results, it is important to consider the possibility that these spiders do exhibit color neophobia, but our experimental methods and design were just unable to uncover it. Here we used prey choice tests with painted termites; our research team has used this same technique, similar full-spectrum lighting, and similar prey choice arenas and protocols to test related hypotheses about how *Habronattus* jumping spiders use color during foraging [36, 38, 39, Ihle and Taylor unpublished data; reviewed above]. All of these previous studies have shown significant responses to color cues. Given this, we can be confident that the spiders should have perceived the color cues we presented to them, and that they could have used these color cues to make decisions. Moreover, the sample sizes used here were comparable to (or larger) than those used in the aforementioned studies. Finally, the non-significant effects in the present study were opposite to those expected from our *a priori* hypotheses. Specifically, we expected spiders to avoid novel-colored prey, but the number of attacks on novel colors was slightly higher (but not significantly so, see Fig 3). Moreover, we expected any effect of novelty to be strongest for the color red, but when the color red was novel, it was attacked more often than the other colors rather than less (again, not significantly so, see Fig 3C). Collectively, this is suggestive of a true negative result (i.e., a true lack of color neophobia in these spiders), although future studies should continue to address questions about color neophobia (e.g., using different prey species, different testing protocols, spiders of different sex/age classes, etc.) that might reveal nuances that were not detected here. Given that our results ran counter to expectation, we may also want to consider the possibility that these spiders exhibit color neophilia (rather than neophobia) [53].

We found no significant main effect of color in our models, suggesting that the spiders did not have robust and inflexible prey color biases that were strong enough to persist through the color exposure period of our experiment. They attacked the three prey colors (blue, green, and red) at equal rates during the choice tests. While examining color biases (independent of neophobia) was not a major goal of the present study, our results here are interesting to think about in the context of what is already known about this species. Previous work with *H*. *pyrrithrix* foraging on artificially colored prey has revealed color biases (with the lowest attack rates on red and yellow prey and the highest attack rates on blue prey) that likely arise from both innate and learned components [34, 36]. We also know from training experiments that these color biases are flexible and can be altered in predictable ways using diet manipulation experiments with colorful prey [31]. This flexibility in these color biases probably explains why, after our 4-week color exposure period (where spiders were exposed repeatedly to two out of the three colors), we didn’t see any evidence of color biases remaining. Any innate color biases were likely extinguished by the 4-week color exposure period, and therefore no longer present at the time of testing. The spiders’ positive experiences with colored termites during the color exposure period should be considered carefully when planning future studies of color neophobia; it may be that the spiders learned that the two familiar-colored termites were palatable, and simply generalized that colored termites in general were not linked to danger dampening any expected effects of neophobia [e.g., see 10].

Jumping spiders and birds both are major visual predators of small insect prey and both of these groups, as well as others, undoubtedly contribute to the evolution of aposematic prey defenses. Our study shows that despite the well-documented similarities between the predator psychology of birds and jumping spiders (reviewed above), there are also key differences. It’s important to continue to understand these differences (from understudied groups) so that we can build a broader and more holistic understanding of the selective pressures that shape colorful prey defenses in nature.
